# Suicide risk and depressive symptoms in obese patients treated with GLP-1 receptor agonists

**DOI:** 10.1192/j.eurpsy.2025.1394

**Published:** 2025-08-26

**Authors:** M. Del Valle Martín, N. Román Avezuela, R. De Hita Santillana, M. L. Costa Ferreira da Silva

**Affiliations:** 1 Psychiatry, Hospital Universitario Severo Ochoa, Leganes; 2 Psychiatry, Clínica San Miguel, Madrid; 3 Psychiatry, Hospital Universitario José Germain, Leganes, Spain

## Abstract

**Introduction:**

The Atlas study, conducted in 2023, shows that the global prevalence of high body mass index (BMI) is expected to increase significantly by 2035, both in adults (from 42% to over 54%) and in young people aged 5-19 years (from 22% to 39%). Also in 2023, the European Medicines Agency (EMA) published a statement warning about the risk of suicidal thoughts and self-harm with glucagon-like peptide-1 receptor agonists (GLP-1 RAs), including semaglutide, tirzepatide and liraglutide. The psychiatric safety of recently developed anti-obesity drugs has not been adequately studied.

**Objectives:**

The objective of this study was to collate and evaluate the existing evidence regarding the psychiatric safety of GLP-1 RAs in individuals without major psychopathology.

**Methods:**

A narrative literature review was carried out in the PubMed, Cochrane and Embase databases, selecting only the articles published in the last 4 years, using the following keywords: depression suicidal ideation, GLP-1 receptor agonists, semaglutide, tirzepatide, liraglutide.

**Results:**

There is a complex relationship between body weight and depressive and anxiety disorders. Obesity can be understood as chronic low-grade inflammation of adipose tissue, and the activation of inflammatory pathways could lead to the development of depression and anxiety. Also, due to cultural norms, obesity contributes to increased body dissatisfaction and lower self-esteem, both of which are associated with depression and anxiety. All of these factors can contribute to the maintenance and worsening of anxiety and depression. In most trials, there was no evidence of an association between treatment with GLP-1 RAs and an increased risk of developing depression or suicidal thoughts/behaviour. Psychiatric adverse events were rare, occurring in only 1.2-1% of patients. Depression was the most common adverse event reported, followed by anxiety and suicidal ideation. Fatal outcomes occurred mainly in men and in people treated with liraglutide, which is associated with a higher risk of suicide than semaglutide. Tagliapietra et al suggest that the patient population receiving GLP-1RAs compared with other diabetes pharmacotherapies is a group with a higher basal risk of developing depression, rather than a causal effect of the drugs. Recent but limited real-world evidence suggests that the use of semaglutide is associated with reduced suicidal ideation and major depression.

**Conclusions:**

The results of our analysis suggest that GLP-1 RAs did not increase the risk of developing symptoms of depression or suicidal thoughts. However, the severity and fatal outcomes of some of these reports warrant further investigation. People with obesity should be monitored for mental health problems so that they can receive appropriate support and care.

**Disclosure of Interest:**

None Declared

